# Simulation of Aging and Bonding Properties of the Matrix/Filler Interface in Particle-Reinforced Composites

**DOI:** 10.3390/polym17111557

**Published:** 2025-06-03

**Authors:** Zebin Chen, Xueren Wang, Zijie Zou, Hongfu Qiang, Xiao Fu

**Affiliations:** 1 Zhijian Laboratory, Rocket Force University of Engineering, Xi’an 710025, China; 13251880807@163.com (Z.C.); 15229233964@163.com (Z.Z.);; 2 School of Astronautics NPU, Northwestern Polytechnic University, Xi’an 710025, China; 3 Chinese People’s Liberation Army Rocket Sergeant School, Weifang 262550, China

**Keywords:** molecular dynamics simulation, adhesive interface, binding energy, particle-reinforced composite

## Abstract

To investigate the microscopic mechanism of aging-induced “dewetting” at the matrix/filler interface in Nitrate Ester Plasticized Polyether (NEPE) propellant, this study decoupled the aging process into two factors: crosslinking density evolution and nitrate ester decomposition. Molecular dynamics (MD) simulations were employed to construct all-component matrix models and matrix/filler interface models with varying aging extents. Key parameters including crosslinking density, mechanical properties, free volume fraction, diffusion coefficients of the matrix, as well as interfacial binding energy and radial distribution function (RDF) were calculated to analyze the effects of both aging factors on “debonding”. The results indicate the following: 1. Increased crosslinking density enhances matrix rigidity, suppresses molecular mobility, and causes interfacial binding energy to initially rise then decline, peaking at 40% crosslinking degree. 2. Progressive nitrate ester decomposition expands free volume within the matrix, improves binder system mobility, and weakens nitrate ester-induced interfacial damage, thereby strengthening hydrogen bonding and van der Waals interactions at the interface. 3. The addition of a small amount of bonding agent improved the interfacial bonding energy but did not change the trend of the bonding energy with aging.

## 1. Introduction

Composite solid propellant is a particle-reinforced composite material, typically composed of polymeric organic compounds as the binder matrix and high-energy fillers such as metal particles and oxidizers. Compared to other types of propellants, it offers advantages including convenient storage and use, low maintenance costs, excellent mechanical properties, and high volumetric specific impulse, making it the mainstream propellant technology today. Since the 1940s, various solid propellants have been successively developed [1,2,3,4,5]. Among them, NEPE propellant, widely adopted for its superior energy output, contains abundant high-energy fillers such as RDX (Cyclotrimethylenetrinitramine), HMX (Cyclotetramethylenetetranitramine), and AP (Ammonium Perchlorate). Its binder matrix features a high nitrate ester content, further enhancing its energetic performance.

Aging adversely affects the service performance of solid propellants. The matrix/filler interface, being a structurally vulnerable region, tends to undergo “dewetting” during aging processes, leading to degraded mechanical properties of the propellant [6]. Consequently, the aging-induced adhesive behavior at this interface has remained a critical research focus in solid propellant studies [7,8].

Due to the thin matrix/filler interface in solid propellants, current experimental characterization means can only indirectly calculate or indirectly determine the adhesive properties of the interface. Experimental characterization means to directly determine the adhesive interface interaction data are still lacking. Molecular dynamics (MD) simulation has emerged as a novel research tool for interfacial studies and has been widely used in the study of interfacial properties.

In studies investigating the bonding strength at matrix/filler interfaces, Chen et al. [9] employed Materials Studio (MS) simulations to compare the binding energies of Al-PBT and AP-PBT interfaces. Their results suggest that larger AP particles exhibit lower binding energy, rendering the AP-PBT interface more prone to debonding. Dong et al. [10] constructed molecular models of HTPB, IPDI, MAPO, and an HTPB/AP interface model, systematically investigating the mechanical behavior of HTPB/AP interfaces under varying strain rates. Their findings demonstrated that interfacial strength increased with strain rate, aligning with the macroscopic mechanical trends observed in propellants. Li et al. [11] combined MD simulations and experiments to explore aging mechanisms in solid propellants. Their interface modeling incorporated both oxidative crosslinking and broken chains in the binder matrix. Simulation results revealed a non-monotonic aging response: the binding energies at AP/HTPB and Al/HTPB interfaces initially increased before decreasing with prolonged aging. In studies of matrix/filler interfacial interactions, Zhang L et al. [12] employed radial distribution function (RDF) analysis and binding energy calculations to investigate the interactions between the GAP-PDMH binder and solid fillers (AP, HMX, and RDX), revealing hydrogen bonding interactions at these interfaces. Lan et al. [13] further examined the binding behavior between GAP-DMH and different RDX crystal facets, demonstrating facet-dependent binding energy in the order (001) > (010) > (100). Their comparative analysis showed that the GAP-DMH/RDX system exhibited enhanced van der Waals interactions and higher binding energy compared to the GAP/RDX interface system. In investigating the bonding agent’s interfacial enhancement mechanisms at matrix/filler interfaces, Zhang P et al. [14,15] conducted MD simulations using MS software (Version 2020) to analyze the adsorption behavior of NPBA (neutral polymer bonding agent) in NEPE propellants. Their simulations revealed strong van der Waals interactions between NPBA’s cyano groups (-CN) and RDX’s nitro groups (-NO_2_), with notably high binding energy at NPBA/RDX interfaces. Feng et al. [16] further explored the functional group effects on HMX/NPBA interactions through MD methods, demonstrating that NPBA-containing -CN functional groups exhibited superior mechanical performance and achieved higher binding energy with HMX compared to other functional group variants.

Current research on matrix/filler interface performance reports the following limitations: (1) insufficient investigation into interfacial property evolution under aging conditions; (2) the aging models were constructed with fewer components, limited changes in model aging, and greater differences from realistic aging; (3) a critical knowledge gap exists regarding the matrix/filler interface aging behavior of NEPE propellants, with a particular failure to elucidate the microscopic mechanisms underlying aging-induced “debonding” in NEPE systems.

Given that the aging of NEPE propellant involves three primary mechanisms—binder system degradation, post-curing, and nitrate ester decomposition [17]—this study focuses on two critical aging pathways: crosslinking density evolution and nitrate ester decomposition. Molecular dynamics (MD) models of the matrix and matrix/filler interfaces were constructed with varying crosslinking densities and nitrate ester decomposition levels. Mechanical properties and molecular diffusion characteristics of the matrix were systematically analyzed, while interfacial binding energy and radial distribution function (RDF) values were calculated for the interface models. Through the analysis of data, the microscopic mechanisms underlying aging-induced “dewetting” at the matrix/filler interface were explored, revealing how structural evolution in the binder matrix compromises interfacial integrity.

## 2. Modeling and Methods

This study employed the molecular dynamics software Materials Studio (Version 2020) for modeling and simulation, utilizing the built-in COMPASS II force field in the software for model optimization. This force field exhibits strong versatility and has been applied in simulation studies of various components of NEPE propellants [15,18,19,20,21,22].

### 2.1. Matrix Modeling

The constructed NEPE propellant matrix model in this study comprises the following components: PEG (polyethylene glycol) as the binder, N-100 (modified hexamethylene diisocyanate crosslinker) as the curing agent, and NG (nitroglycerin) and BTTN (1,2,4-butanetriol trinitrate) as plasticizers, with NPBA serving as the interfacial modifier [22]. The molecular model is illustrated in Figure 4. The PEG polymer chains were constructed with a polymerization degree of 90, corresponding to a molecular weight of ~4000 Da, which exhibited natural coiling conformation after geometric optimization. Following typical NEPE formulation mass ratios [1], the model incorporates 200 NG molecules and 200 BTTN molecules, along with 12 PEG chains and 8 N-100 molecules at a curing ratio R = 1. For systems containing bonding agents, two additional geometrically optimized NPBA chains were incorporated. The temperature condition was set to 298 K. The initial density was calculated to be 1.45 g·cm^−3^ based on the mass ratio of the added components, and the initial model size was 50 × 50 × 66.6 Å^3^.

#### 2.1.1. Modeling of Matrix Models for Different Crosslink Densities

**Figure 1:**
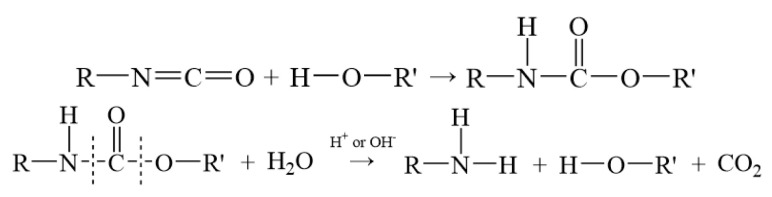
Chemical equation for oxidative crosslink and degradation chain-breaking reaction.

Solid propellant aging induces competing chemical processes in the binder system—oxidative crosslinking (increasing crosslink density) and chain scission degradation (decreasing crosslink density) [23]. The change in crosslink density of NEPE propellant is divided into two distinct segments [24]: minimal crosslink density fluctuation occurs prior to stabilizer depletion, indicating dynamic equilibrium between crosslinking and degradation reactions; Post-stabilizer exhaustion, acidic byproducts from nitrate ester decomposition preferentially drive binder chain scission, triggering rapid crosslink density decline. The mechanistic interplay between these oxidative crosslinking and chain scission pathways in NEPE propellants is schematically detailed in Figure 1 [25].

**Figure 2:**
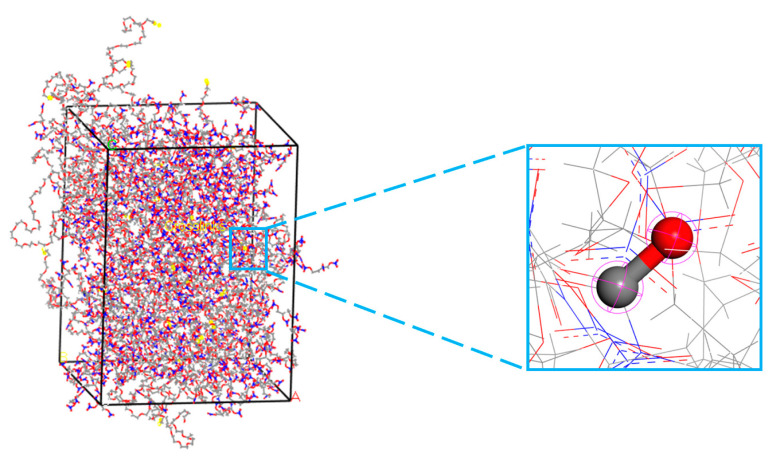
Crosslink points in the model.

Following the crosslinking reaction mechanisms, hydroxyl (-OH) groups on PEG chains were stochastically crosslinked with isocyanate (-NCO) groups of the N-100 curing agent within the matrix model [26]. Four matrix models with distinct crosslinking degrees (20%, 40%, 60%, and 80%) were constructed. The crosslinking reaction temperature was set at 350 K [27]. The crosslinking points in the model are shown in Figure 2.

#### 2.1.2. Modeling of Matrix Models for Different Nitrate Decomposition Degrees

Prolonged aging significantly accelerates nitrate ester decomposition in solid propellants [28]. In NEPE propellants, the commonly used nitrate ester plasticizers—nitroglycerin (NG) and 1,2,4-butanetriol trinitrate (BTTN)—exhibit complex thermal decomposition pathways involving multiple intermediate species and transient states [29,30]. This study focuses exclusively on the thermodynamically stable decomposition products of these nitrate esters. In energetic materials, the nitro group is considered the weakest chemical bond, and the stability of the nitrate group is reduced by the presence of adjacent nitro groups. Therefore, the first decomposition step of nitrate esters is typically denitration. Subsequently, based on the bond energy strength of nitrate esters, decomposition generates substances such as formaldehyde (CH_2_O), nitrogen dioxide (NO_2_), and nitrous acid (HONO). Formaldehyde is oxidized by nitrogen dioxide to produce gases including CO, CO_2_, NO, H_2_O, and other things (specific reaction equations and gas composition ratios can be found in Reference [31]). The NO_2_ generated during the initial decomposition phase is absorbed by the stabilizer. After the stabilizer is depleted, a portion of the reaction-produced gases is released, while the remainder remains within the propellant. Based on the nitrate ester decomposition and propellant weight loss patterns described in Reference [32], it is assumed that 50% of the generated gases are retained within the propellant.

**Figure 3:**
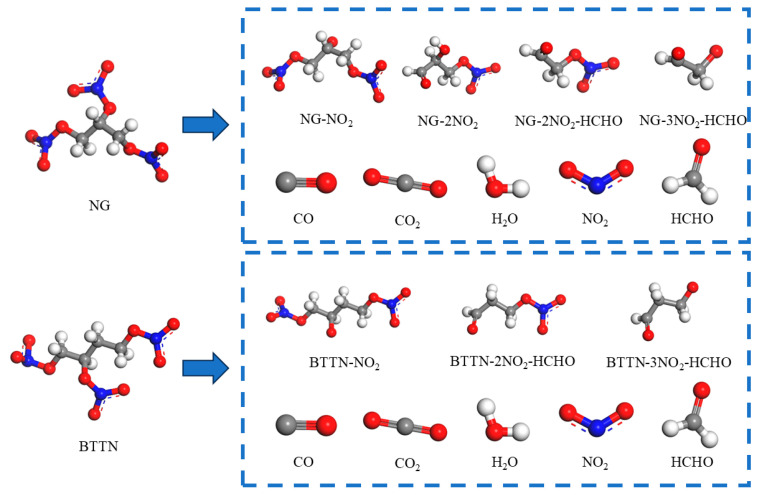
Molecular model of nitrates and their decomposition products.

When establishing the matrix models for varying degrees of nitrate ester decomposition, the decomposition of NG and BTTN is divided into three stages. Primary decomposition of NG involves denitration, with the de-nitro product denoted as NG-NO_2_; secondary decomposition removes either two nitro groups or one nitro group plus one formaldehyde, yielding products labeled NG-2NO_2_ and NG-2NO_2_-HCHO; and tertiary decomposition eliminates three nitro groups and one formaldehyde, resulting in NG-3NO_2_-HCHO. BTTN follows analogous decomposition pathways: primary product: BTTN-NO_2_; secondary product: BTTN-2NO_2_-HCHO; and tertiary product: BTTN-3NO_2_-HCHO. The molecular models of nitrate esters and their decomposition products are illustrated in Figure 3.

When constructing aged matrix models with nitrate ester decomposition degrees of 20%, 40%, 60%, and 80%, the crosslinking degree was uniformly set to 20% while keeping other parameters constant. This design facilitates controlled variable comparison and more directly reflects the impact of nitrate ester decomposition on interfacial properties. According to the principles and patterns of nitrate ester decomposition, the additive components must differ across models with varying decomposition degrees. The specific compositions added to each model are detailed in Table 1 and Table 2.

### 2.2. Filler Modeling

**Figure 4:**
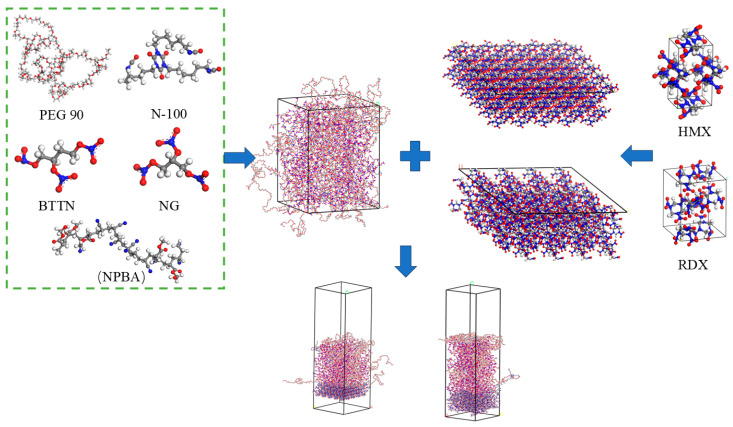
The interface model construction process.

In NEPE propellants, the primary oxidizer fillers are HMX and RDX, accounting for approximately 40–50% of the propellant mass [33]. Using the HMX and RDX crystal models from References [34,35], the HMX (1 0 1) crystal facet (lattice parameters: a = 43.78 Å, b = 41.34 Å, γ = 90°) and RDX (2 1 0) crystal facet (a = 40.87 Å, b = 52.5 Å, γ = 90°) [36,37], which exhibit stable cleavage properties, were selected. A 4-layer thickness was cut for both facets. Subsequently, the HMX (1 0 1) and RDX (2 1 0) facets were expanded in the *x*- and *y*-axis directions by factors of 5 × 5 and 4 × 2, respectively, to match the size of the matrix model. During the expansion process, the vacuum layer thickness was set to 0 Å to facilitate the construction of the interfacial model in the next step. The crystal models and their supercell models are shown in Figure 4.

### 2.3. Interface Modeling

The interface model was constructed with the oxidizer fillers HMX and RDX as the first layer, and the matrix models with varying crosslinking degrees and nitrate ester decomposition levels as the second layer. The orientation from the first layer was used and a 40 Å vacuum layer was added above the matrix to eliminate disruptions caused by periodic boundary conditions along the *z*-axis. The interface model construction process is illustrated in Figure 4.

### 2.4. Model Optimization

**Figure 5:**
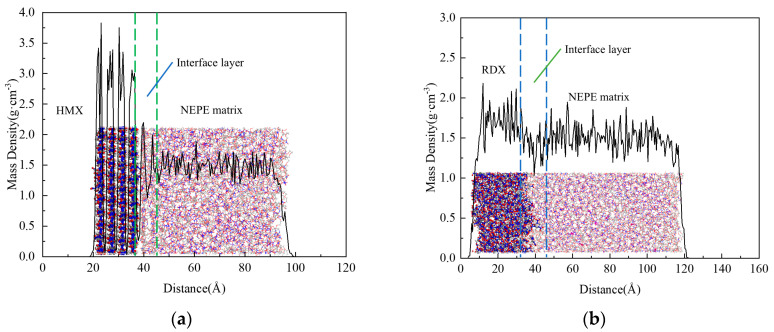
The interface model density distribution: (**a**) matrix/HMX interface; (**b**) matrix/RDX interface.

The NEPE propellant matrix model and matrix/filler interface model underwent geometry optimization using the smart minimizer method with a maximum of 5000 iterations. Subsequently, molecular dynamics equilibrium calculations were performed under the NPT ensemble for 1 ns with an applied pressure of 1 kPa. This was followed by annealing treatment from 298 K to 800 K for 10 cycles within the same ensemble. After annealing, the system was re-equilibrated under the NPT ensemble, then further equilibrated under the NVT ensemble for 1 ns, and finally subjected to additional geometry optimization with up to 5000 iterations. At this stage, the temperature and energy curves stabilized, indicating that the model had reached equilibrium. During relaxation, the NEPE matrix spontaneously adsorbed and formed a stable bond with the solid fillers. The mass density profile along the *z*-axis of the interface model is shown in Figure 5. As illustrated, the density curves of the two oxidizer-based interface models are divided into three distinct segments after molecular dynamics equilibration: the first segment with higher density corresponds to the oxidizer fillers; the third segment with lower density represents the NEPE matrix; and the intermediate transition zone between them constitutes the matrix/filler interface. The interfacial thickness is approximately 9 Å for the matrix/HMX model and 13 Å for the matrix/RDX model. This confirms adhesive bonding between the NEPE matrix and both oxidizer fillers, validating the models for subsequent computational analysis.

## 3. Results and Discussion

### 3.1. Changes in Matrix Properties After Aging

The matrix is the key area of aging in solid propellants, exhibiting significant changes in properties during the aging process, which serves as a critical factor leading to variations in matrix/filler interface performance with aging. The analysis of matrix property changes helps to further explore the microscopic mechanisms underlying the interface performance evolution between the matrix and filler.

#### 3.1.1. Characterization Data of Matrix Properties

In this paper, the matrix properties were characterized by mechanical properties, free volume fraction, and the diffusion coefficient.

Through computational analysis of the mechanical properties of the matrix model, one can gain an intuitive understanding of the matrix’s performance variations. Simulation calculations yielded mechanical parameters such as elastic constants, Young’s modulus (E), bulk modulus (K), shear modulus (G), Poisson’s ratio (υ), and Lamé constants (Lambda, Mu). Due to the small scale of the constructed model, the simulation data of Young’s modulus and Poisson’s ratio have slightly different values in each direction, so the average values are taken to represent them. The bulk modulus and shear modulus were calculated using the Hill model.

Free volume refers to the unoccupied space remaining after subtracting the actual molecular volume (van der Waals volume or intrinsic volume) from the total volume of a material. It significantly influences the glass transition temperature, gas permeability, and diffusion rates in polymeric materials, serving as a preliminary indicator of the mobility of internal components. The free volume fraction (FFV) is defined by the following formula [18]:

(1) $$\mathrm{FFV}=\frac{V_{F}}{V_{F}+V_{O}}$$

where $V_{F}$ is the free volume within the polymer; $V_{O}$ is the volume occupied by the molecular chain.

The diffusion coefficient (*D*) serves as the fundamental physical quantity characterizing material diffusivity, directly quantifying the transport rate per unit area under a unit concentration gradient. Molecular dynamics simulations were conducted on the matrix model under NVT ensemble conditions for 150 ps. Mean square displacement (MSD) data of the model were obtained through calculations in the Dynamic module, from which the diffusion coefficient was derived using the following formula [38]:

(2) $$D=\underset{t\to \infty }{\lim}\frac{\mathrm{MSD}}{6t}=\frac{m}{6}$$

where m is the slope after stabilization of the curve plotted with time t as the *x*-axis and MSD as the *y*-axis. To ensure comparative rationality, the diffusion coefficients of NG and BTTN (D_NG_, D_BTTN_) were calculated for matrix models with different crosslinking degrees for comparison, while the diffusion coefficient of PEG (D_PEG_) was evaluated for matrix models with varying decomposition levels through computation, thereby enabling a systematic comparative analysis.

#### 3.1.2. Effect of Crosslinking Degree Variation on Matrix Properties

**Figure 6:**
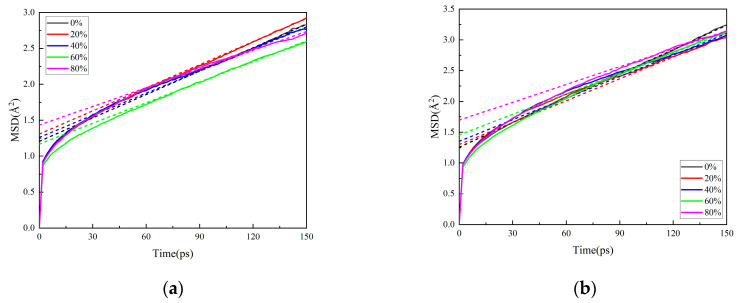
The mean square displacement curve of the matrix model with different degrees of crosslinking: (**a**) NG; (**b**) BTTN.

With variations in crosslinking degree, the mechanical property parameters of the matrix are summarized in Table 3 and Table 4, while the free volume fraction is tabulated in Table 5. The mean square displacement (MSD) curves are shown in Figure 6. The dashed line in the figure represents the linear fit to the slope of the MSD curves after stabilization, i.e., the diffusion coefficient, with specific values provided in Table 6.

As the degree of crosslinking increases, the elastic modulus, bulk modulus, and shear modulus increase, while the Poisson’s ratio and K/G ratio decrease. This indicates that the three-dimensional network of the crosslinked matrix becomes more compact, resulting in greater hardness, reduced ductility, enhanced rigidity, improved deformation resistance, and increased shear resistance. However, these changes also introduce a certain risk of embrittlement.

The total volume and free volume fraction of the polymer decrease with increasing crosslinking degree, indicating that the formation of crosslinked networks enhances the tight packing of molecular chains and induces structural tightening of the matrix, which aligns with the mechanical property calculation results. Consequently, the available space for molecular movement within the matrix is reduced, leading to increased restrictions on various molecular motions. The volume of the matrix model shows a modest reduction with higher crosslinking degrees, demonstrating that oxidative crosslinking reactions induce an overall tightening of the internal structure, although the extent of tightening remains limited.

The slopes of the MSD curves for NG and BTTN gradually decrease with increasing crosslinking degree, accompanied by reduced diffusion coefficients. This indicates that the formation of a three-dimensional crosslinked network leads to a refined internal matrix organization, hindering the motion of both NG and BTTN molecules. Similarly, the movement of PEG long chains becomes significantly more restricted under these conditions.

#### 3.1.3. Effect of Nitrate Ester Decomposition on Matrix Properties

**Figure 7:**
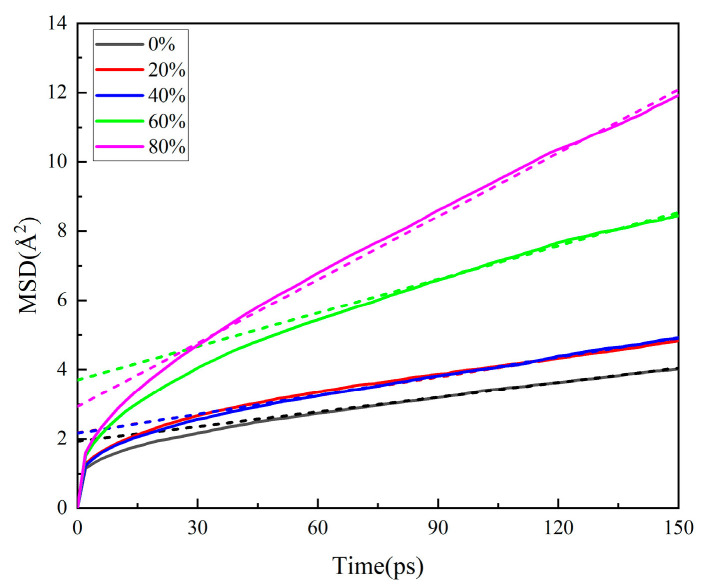
The MSD of different nitric ester decomposition matrix models.

The mechanical property parameters of the matrix under nitrate ester decomposition conditions are detailed in Table 7 and Table 8, with the free volume fraction presented in Table 9. The MSD curves are shown in Figure 7. The slope of the dashed line corresponds to the diffusion coefficient, with specific values provided in Table 10.

As the degree of nitrate ester decomposition intensifies, the mechanical properties of solid propellants demonstrate an inverse trend compared to the effects of increased crosslinking degree, with degraded mechanical performance and progressive material softening. This trend is corroborated by the observed variations in elastic constants.

The free volume fraction increases with the intensification of nitrate ester decomposition, due to cavities generated by decomposition contributing additional free volume. This creates enhanced molecular mobility as the matrix gains more spatial freedom for molecular movement. Concurrently, nitrate ester decomposition induces substantial volumetric changes in the matrix, with a portion of the generated gas escaping from the system, resulting in a measurable reduction in the matrix model volume.

The diffusion coefficients of PEG chains exhibit a significant increase with intensified nitrate ester decomposition. Extensive decomposition of NG and BTTN leads to a reduction in molecular weight within the matrix, weakening intermolecular interactions with PEG chains and diminishing steric hindrance effects. Concurrently, the retention of decomposition-generated gases within the matrix creates expanded motional freedom for PEG chains. These combined effects enhance the mobility of the binder system. This trend aligns with the free volume fraction (FFV) computational conclusions.

### 3.2. Changes in Interface Properties After Aging

#### 3.2.1. Characterization Data of Matrix/Filler Interface Properties

This study systematically investigates interfacial performance evolution through two characterization methodologies: interfacial adhesion energy and radial distribution function (RDF) analysis. By integrating matrix aging performance evaluations, the research conducts a comparative investigation on the effects of crosslinking density variations and nitrate ester decomposition on interfacial properties.

Interfacial adhesion energy serves as a critical parameter for characterizing interfacial strength. A higher interfacial adhesion energy value indicates stronger interfacial bonding integrity and greater resistance to mechanical failure. The interfacial adhesion energy can be calculated using the following formula [39]:

(3) $$E_{\mathrm{bind}}=-E_{\mathrm{int}\mathrm{er}}=-(E_{total}-E_{matrix}-E_{partical})$$

where $E_{\mathrm{int}\mathrm{er}}$ is the interaction energy of the interface, $E_{total}$ is the total energy of the constructed interface model, $E_{matrix}$ is the total energy of the matrix model in the interface, and $E_{partical}$ is the total energy of the solid oxidizer filler model of the interface. The specific calculated values of the binding energy in this work are provided in Table A1 and Table A2.

The radial distribution function (g(r)) quantifies the ratio of the probability density of finding another molecule at a distance r from a central molecule to the probability density expected in a completely random distribution. This function serves as a critical indicator of intermolecular interaction strength, enabling the detection of hydrogen bonding and van der Waals forces at interfaces through analysis of peak positions and profile characteristics in the g(r) curve [40]. The mathematical expression is defined as follows:

(4) $$g(r)=\frac{n(r)}{\rho \cdot 4\pi r^{2}\Delta r}$$

where n(r) is the average number of particles within a shell of width Δr at a distance r from the reference particle, ρ is the number density of atoms, and $4\pi r^{2}\Delta r$ is the volume of the shell. When the value of r falls within 2.6–3.2 Å, it indicates that the intermolecular forces correspond to hydrogen bonding interactions. For values between 3.2 and 5.0 Å, the observed forces represent strong Van der Waals interactions, while values exceeding 5.0 Å suggest weak Van der Waals interactions. The radial distribution function curves presented in this study were smoothed using a fourth-order polynomial fitting method.

#### 3.2.2. Effect of Crosslinking Degree Changes on Interface Properties

**Figure 8:**
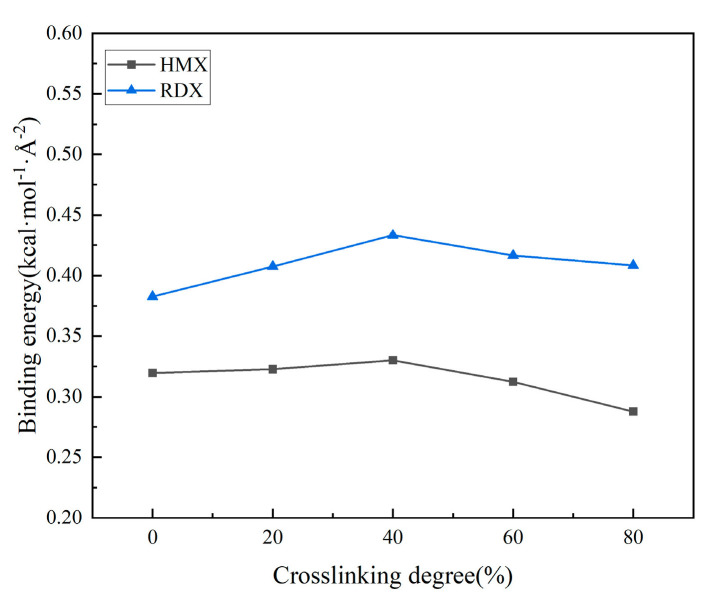
The interface model binding energies at different degrees of crosslinking.

**Figure 9:**
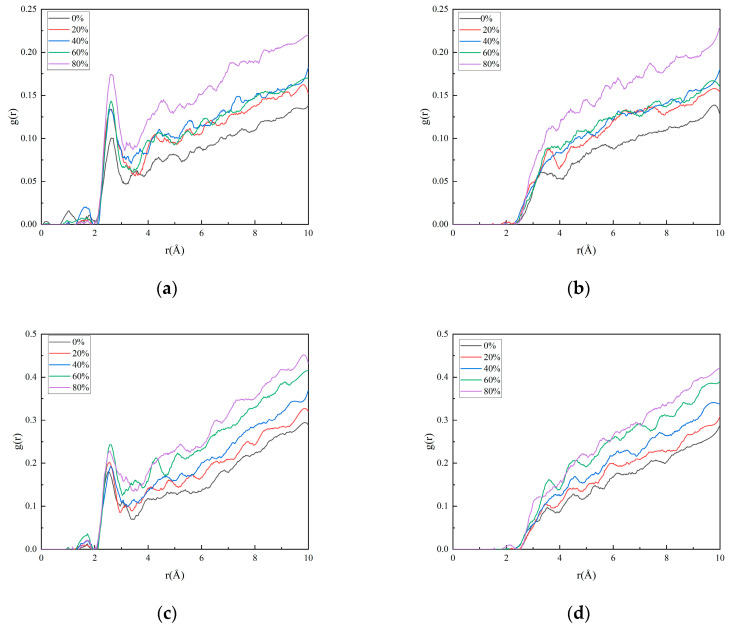
The radial distribution function of the interface model with different degrees of crosslinking: (**a**) H_m_-O_HMX_; (**b**) H_m_-N_HMX_; (**c**) H_m_-O_RDX_; and (**d**) H_m_-N_RDX_.

Figure 8 illustrates the evolution of binding energy between the matrix and filler under different crosslinking densities, while Figure 9 displays the radial distribution function (RDF) curves of the interface at varying crosslinking densities. In the matrix, hydrogen atoms are labeled as H_m_, oxygen atoms in HMX and RDX are denoted as O_HMX_ and O_RDX_, respectively, and nitrogen atoms in HMX and RDX are represented as N_HMX_ and N_RDX_.

**Figure 10:**
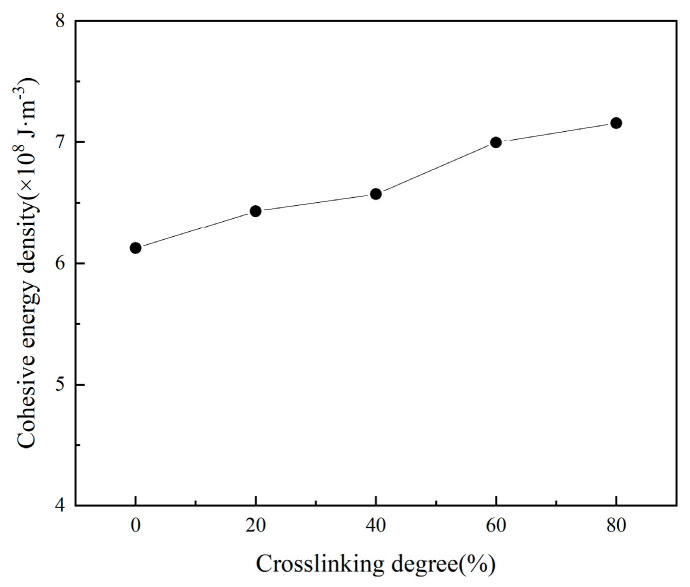
The cohesive energy in a matrix model with different degrees of crosslinking.

From the binding energy curve, it can be observed that the binding energy initially increases and then decreases with the degree of crosslinking. Among the five tested crosslinking densities, the binding energy reaches its maximum value at a crosslinking density of 40%, while both insufficient and excessive crosslinking densities result in lower binding energy. The analysis suggests that at lower crosslinking densities, the matrix contains fewer crosslinking points, resulting in a loose three-dimensional network structure and short adhesive molecular chains. This configuration allows easy sliding and high segmental mobility, leading to strong matrix fluidity but poor viscosity. Consequently, insufficient interfacial bonding forms between the matrix and solid fillers, resulting in suboptimal adhesive performance. Conversely, excessive crosslinking causes molecular chains to become excessively constrained. Analysis of the matrix properties reveals that under such conditions, the matrix hardness increases significantly with limited free volume, causing deteriorated molecular diffusivity. This restricted molecular chain mobility hinders the migration of adhesive system molecules to the interface. Moreover, the cohesive energy of the matrix increases with higher crosslinking degrees (Figure 10), causing the adhesive system to contract. This creates additional accommodation space for nitrate esters at the interface, which prevents sufficient wetting between the adhesive system and solid fillers during bonding. Consequently, the matrix bonding effectiveness deteriorates.

The radial distribution function (RDF) plots reveal that the peak position between hydrogen (H) atoms in the matrix and oxygen (O) atoms in the oxidizer filler lies within 2.6–2.7 Å, indicating the formation of hydrogen bonding interactions. In contrast, the peak between H atoms in the matrix and nitrogen (N) atoms in the oxidizer filler appears at 3.5–3.7 Å with no prominent peak intensity; this indicates that no hydrogen bonds are formed between them, with only stronger van der Waals interactions present. The bonding effectiveness of the matrix toward both fillers follows a similar trend. As the crosslinking density increases, the radial distribution function (RDF) peaks intensify, and the post-peak curve profile rises, indicating enhanced hydrogen bonding and van der Waals interactions at the interface. This enhancement implies stronger interfacial interactions at effective contact regions between the matrix and filler. However, when the crosslinking density exceeds an optimal threshold, while the RDF values increase, the binding energy decreases. This apparent contradiction arises because excessive crosslinking reduces the effective contact area between the adhesive system and the oxidizer filler, despite the intensified localized interactions. Across the entire range of crosslinking densities, the binding energy and radial distribution function (RDF) values of RDX under identical conditions are consistently higher than those of HMX. This disparity is attributed to the smaller ring size of RDX.

#### 3.2.3. Effect of Nitric Acid Ester Decomposition on Interface Properties

**Figure 11:**
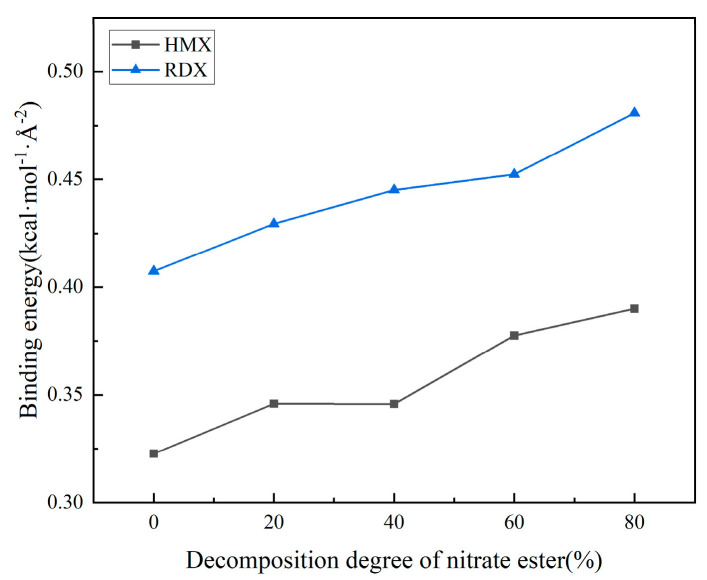
The binding energies of the interface model for different nitrate decomposition degrees.

**Figure 12:**
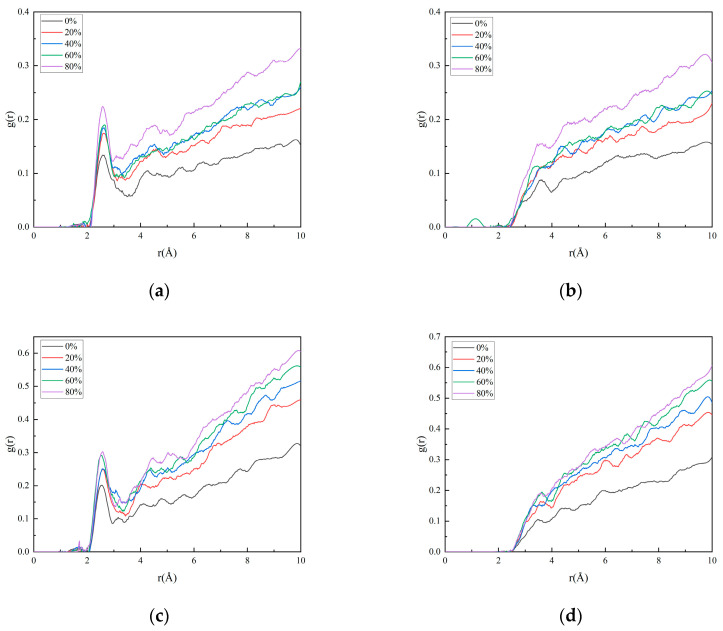
The radial distribution function of the interface model for different nitrate decomposition degrees: (**a**) H_m_-O_HMX_; (**b**) H_m_-N_HMX_; (**c**) H_m_-O_RDX_; and (**d**) H_m_-N_RDX_.

Figure 11 and Figure 12 illustrate the evolution of binding energy and radial distribution function (RDF) for the matrix/filler interface model under varying degrees of nitrate ester decomposition.

From the curve depicting the binding energy versus the nitrate ester decomposition degree, it can be observed that as the degree of nitrate ester decomposition increases, the interfacial binding energy gradually rises. Concurrently, the radial distribution function (RDF) curves reveal that the hydrogen bonding interactions (H_m_-N_HMX_, H_m_-N_RDX_) and van der Waals interactions (H_m_-N_HMX_, H_m_-N_RDX_) also strengthen with intensified nitrate ester decomposition. These consistent trends in both datasets confirm that interfacial interactions are progressively enhanced as decomposition advances.

The nitrate ester plasticizers added to solid propellants are oily substances [41]. When nitrate esters remain at the matrix/filler interface, they exert detrimental effects on interfacial bonding. After the decomposition of nitrate esters, their content at the matrix/filler interface decreases, thereby reducing the interfacial damage caused by nitrate esters. From the analysis of matrix aging performance, it is evident that after the decomposition of nitrate esters, the fluidity of the matrix is enhanced, resulting in improved wetting of the oxidizer filler. Additionally, the decomposition process creates larger free volume and mobility space for molecular chains in the adhesive system, enabling them to migrate more readily to the interface. This facilitates closer interfacial contact with the oxidizer filler, thereby strengthening hydrogen bonding and van der Waals interactions and ultimately improving bonding performance. Comparative analysis with simulations of varying crosslinking density models reveals that the interfacial performance enhancement after nitrate ester decomposition is more pronounced, highlighting the dominant role of nitrate ester decomposition in governing interfacial properties.

The intensified decomposition of nitrate esters indicates more severe aging, and most experimental results demonstrate that prolonged aging time leads to deterioration in interfacial properties [42,43,44]. The analysis attributes this degradation to gas accumulation at the interface resulting from nitrate ester decomposition. These gases form air pockets within the interfacial region, which reduce the effective contact area between the matrix and filler, leading to significant deterioration in bonding performance.

#### 3.2.4. The Resistance of Bonding Agents to Aging

**Figure 13:**
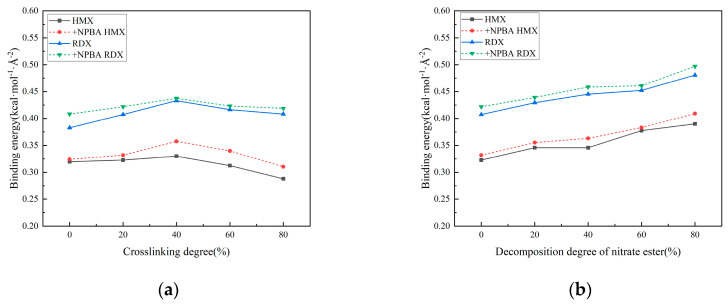
Binding energy curves of the interface models after the addition of the bonding agent: (**a**) interface model of different crosslinking densities; (**b**) interface model of different nitrate ester decomposing degrees.

The binding energy curves corresponding to varying crosslinking densities and different degrees of nitrate ester decomposition after bonding agent addition are presented in Figure 13.

Bonding agents are incorporated into the adhesive system through chemical reactions with curing agents. Subsequently migrating to the interface, they establish stronger interactions with oxidizer particles while simultaneously forming a protective film on the filler surfaces, thereby preventing nitrate esters from causing interfacial damage [14,15,16]. As evidenced by the two figures, the interfacial binding energy increases upon the incorporation of the NPBA bonding agent, suggesting its reinforcement effect at the matrix/filler interface. However, owing to the insufficient dosage, the enhancement effect remains marginal. The variation trend of binding energy after bonding agent addition aligns with that of the non-additive interfacial model. Similarly, it first increases and then decreases with crosslinking density, while increasing with the intensification of nitrate ester decomposition. This indicates that insufficient bonding agent dosage results in incomplete protective layer formation on the filler surface. Although interfacial adhesion is improved, it cannot mitigate the aging-induced degradation of interfacial properties caused by crosslinking density changes and nitrate ester decomposition. Within the full range of crosslinking and nitrate ester decomposition, the binding energy follows the order of NPBA-RDX > RDX > NPBA-HMX > HMX.

## 4. Conclusions

To investigate the microscopic mechanism of aging-induced “dewetting” at the NEPE propellant matrix/filler interface, this study decouples the aging process into two components: crosslinking density variations and nitrate ester decomposition. Matrix and matrix/filler interface models under different aging conditions were constructed to analyze the microscopic causes of interfacial property degradation during aging. The key conclusions are as follows:

(1) The increased crosslinking density of the matrix results in structural contraction and reduced ductility. Elevated crosslinking reduces the free volume fraction and diffusion coefficient within the matrix, intensifies hindrance to molecular chain movement, and suppresses the mobility of polyethylene glycol (PEG) chains. The interfacial binding energy first increases and then decreases with crosslinking density, peaking at 40% crosslinking. The analysis suggests that at low crosslinking densities, the strong fluidity and weak viscosity of the molecular chains of adhesives prevent sufficient bonding with solid fillers. Conversely, excessive crosslinking severely restricts molecular chain mobility, and the binder system exhibits high cohesive energy, hindering the migration of the adhesive system to the matrix/filler interface and resulting in inadequate interfacial wetting. Notably, hydrogen bonding interactions and van der Waals forces continuously strengthen with crosslinking density, contrasting with the non-monotonic trend of binding energy. This discrepancy arises from the reduced effective contact area between the adhesive system and oxidized fillers under high crosslinking conditions.
(2) As the degree of nitrate ester decomposition intensifies, the evolution of mechanical properties in the solid propellant exhibits a trend opposite to that induced by increased crosslinking density. Following decomposition, the matrix shows expanded free volume, enhanced diffusivity, and progressively improved molecular chain mobility. The interfacial binding energy increases with intensified nitrate ester decomposition, accompanied by strengthened hydrogen bonding and van der Waals interactions, resulting in significant improvement in interfacial performance. The analysis reveals that nitrate ester decomposition reduces their interfacial concentration, thereby mitigating their detrimental effects on the interface. Concurrently, the decomposition generates additional free volume and molecular mobility, facilitating adhesive system migration to the matrix/filler interface. This promotes enhanced contact between the binder and oxidizer filler particles.
(3) The addition of a small amount of bonding agent modestly increases interfacial binding energy. However, it fails to alter the trend of interfacial binding energy variation with crosslinking density and nitrate ester decomposition. This serves as evidence that insufficient bonding agent dosage cannot counteract the adverse effects of these two factors on interfacial performance.

The above conclusions provide further insights into the aging-induced variations in matrix/filler interfacial properties, establishing a theoretical foundation for mitigating the aging-related “dewetting” issue in NEPE propellants. To enhance the interfacial performance in subsequent studies, the crosslinking density should be maintained within an appropriate range (avoiding excessive or insufficient levels). Additionally, the addition of nitrate esters and bonding agents must be controlled within reasonable limits, while migration of nitrate esters towards the matrix/filler interface should be prevented.

## Figures and Tables

**Table 1:** The addition of NG and its decomposition products in different models.

Decomposition Degree of Nitrate Ester/%	NG	NG-NO_2_	NG-2NO_2_	NG-2NO_2_-HCHO	NG-3NO_2_-HCHO	CO	CO_2_	H_2_O	NO_2_	HCHO
0	200	0	0	0	0	0	0	0	0	0
20	160	40	0	0	0	0	0	0	0	0
40	120	60	0	20	0	0	0	0	0	20
60	80	60	20	40	0	12	8	16	42	14
80	40	80	20	40	20	21	14	28	60	25

**Table 2:** The addition of BTTN and its decomposition products in different models.

Decomposition Degree of Nitrate Ester/%	BTTN	BTTN-NO_2_	BTTN-2NO_2_-HCHO	BTTN-3NO_2_-HCHO	CO	CO_2_	H_2_O	NO_2_	HCHO
0	200	0	0	0	0	0	0	0	0
20	160	40	0	0	0	0	0	0	0
40	120	60	20	0	0	0	0	0	20
60	80	80	40	0	12	8	16	42	14
80	40	80	60	20	21	14	28	60	25

**Table 3:** The elastic constants of matrix models with different degrees of crosslinking.

Crosslinking Degree/%	C11	C12	C13	C22	C23	C33	C44	C55	C66
0	5.5922	2.376	2.5047	5.4498	2.6745	5.4005	1.7217	1.6347	1.5344
20	5.6271	2.3821	2.2249	5.7568	2.7175	4.9757	1.6195	1.5192	1.4276
40	5.5701	2.4311	2.3333	5.6508	2.4019	5.842	1.6734	1.4959	1.688
60	5.7513	2.6488	2.5833	6.0666	2.5824	6.0417	1.6772	1.7863	1.7603
80	6.2882	2.7672	2.6226	6.1274	2.6904	5.8225	1.6418	1.8112	1.6814

**Table 4:** The mechanical property parameters of matrix models with different degrees of crosslinking.

Crosslinking Degree/%	E/Gpa	K/Gpa	G/Gpa	υ	K/G	Lambda/Gpa	Mu/Gpa
0	3.866	3.5023	1.4182	0.3169	2.469539	2.2203	1.6303
20	3.8834	3.4178	1.5625	0.3108	2.187392	2.6756	1.3888
40	4.2356	3.4676	1.617	0.3067	2.144465	2.4495	1.6191
60	4.3607	3.7174	1.7398	0.3047	2.136682	2.4707	1.7413
80	4.4117	3.8085	1.79	0.2954	2.127654	2.6564	1.7115

**Table 5:** The free volume fraction of matrix models with different degrees of crosslinking.

Crosslinking Degree/%	0	20	40	60	80
Occupied Volume/Å^3^	155,875.53	155,832.2	155,959.6	156,242.12	156,674.17
Free Volume/Å^3^	16,899.84	16,360.2	16,157.33	16,136.83	15,909.09
FFV	0.097	0.095	0.094	0.093	0.092

**Table 6:** The diffusion coefficients of matrix models with different degrees of crosslinking.

Crosslinking/Decomposition Degree	0%	20%	40%	60%	80%
D_NG_	0.00181	0.00179	0.00171	0.00159	0.00145
D_BTTN_	0.00220	0.00197	0.00195	0.00185	0.00161

**Table 7:** The elastic constants of different nitric ester decomposition matrix models.

Decomposition Degree of Nitrate Ester/%	C11	C12	C13	C22	C23	C33	C44	C55	C66
0	5.6271	2.3821	2.2249	5.7568	2.7175	4.9757	1.6195	1.5192	1.4276
20	5.392	2.0663	2.3154	5.2	2.3105	5.5194	1.5921	1.6272	1.3703
40	5.3344	2.5451	2.4526	5.4083	2.4201	5.3814	1.6868	1.6208	1.6521
60	4.4232	1.8355	1.7624	4.5491	1.834	4.2122	1.2992	1.2405	1.3938
80	3.6417	1.4658	1.5669	3.8825	1.3367	3.2762	1.0447	1.078	1.0731

**Table 8:** The mechanical property data of different nitric ester decomposition matrix models.

Decomposition Degree of Nitrate Ester/%	E/Gpa	K/Gpa	G/Gpa	υ	K/G	Lambda/Gpa	Mu/Gpa
0	3.8834	3.4178	1.5625	0.3108	2.187392	2.6756	1.3888
20	4.0376	3.2691	1.5366	0.2937	2.127489	2.3107	1.5299
40	3.8089	3.4322	1.5674	0.27	2.189741	2.0682	1.6532
60	3.331	2.6695	1.301	0.2923	2.051883	1.7725	1.3112
80	2.6925	2.1489	1.0519	0.2908	2.042875	1.4696	1.0653

**Table 9:** The free volume of different nitric ester decomposition matrix models.

Decomposition Degree of Nitrate Ester/%	0	20	40	60	80
Occupied Volume/Å^3^	155,832.2	153,799.28	149,102.1	149,852.12	146,233.77
Free Volume/Å^3^	16,360.2	16,730.34	17,083.08	19,224.23	19,503.03
FFV	0.095	0.098	0.103	0.114	0.118

**Table 10:** The diffuse coefficients of different nitric acid ester decomposition matrix models.

Decomposition Degree/%	0	20	40	60	80
D_PEG_	0.00235	0.00299	0.00305	0.00538	0.01017

## Data Availability

The original contributions presented in this study are included in the article. Further inquiries can be directed to the corresponding authors.

## Abbreviations

The following abbreviations are used in this manuscript:

NEPE	Nitrate Ester Plasticized Polyether
MD	Molecular dynamics
RDF	Radial distribution function
RDX	Cyclotrimethylenetrinitramine
HMX	Cyclotetramethylenetetranitramine
AP	Ammonium Perchlorate
MS	Materials Studio
GAP	Glycidyl azide polymer
PDMH	3-propenyl-5,5-Dimethylhydantoin
DMH	Dimethyl hydantoin
IPDI	Isophorone diisocyanate
MAPO	Methyl-aziridinyl phosphine oxide
NPBA	Neutral polymer bonding agent
PEG	Polyethylene glycol
NG	Nitroglycerin
BTTN	1,2,4-butanetriol trinitrate
N-100	Modified hexamethylene diisocyanate crosslinker
NPT	Constant-pressure, constant-temperature ensemble
NVT	Canonical ensemble
FFV	Free volume fraction
MSD	Mean square displacement

## Appendix A

**Table A1:** The detailed calculated values of interfacial binding energy for different crosslinking degrees.

Interface Type	Crosslinking Density/%	*E_total_*/kcal·mol^−1^	*E_matrix_*/kcal·mol^−1^	*E_partical_*/kcal·mol^−1^	*E*_bind_/kcal·mol^−1^	Interface Area/Å^2^	Binding Energy/kcal·mol^−1^·Å^−2^
HMX	0	−90,623.17098	−38,219.10492	−51,487.20119	−916.86486	2868.6736	−0.31961282
	20	−90,890.28969	−38,460.5983	−51,495.5587	−934.132689	2894.44	−0.322733478
	40	−91,087.35064	−38,666.64236	−51,466.18415	−954.524139	2892.2884	−0.330023845
	60	−91,388.90353	−39,047.69977	−51,441.9902	−899.213565	2877.786	−0.312467141
	80	−91,391.53002	−39,215.59974	−51,348.8544	−827.075879	2874.0321	−0.287775449
+NPBA HMX	0	−90,893.87013	−38,527.73516	−51,438.2732	−927.861775	2862.25	−0.324172164
	20	−91,156.51333	−38,802.71472	−51,397.30879	−956.489827	2884.7641	−0.331566046
	40	−91,152.43717	−38,833.12877	−51,293.30148	−1026.00693	2868.6736	−0.357659
	60	−91,216.94157	−39,042.93097	−51,205.2364	−968.774201	2854.7649	−0.339353409
	80	−91,308.84627	−39,271.72943	−51,145.05954	−892.057295	2872.96	−0.310501119
RDX	0	−89,654.5332	−38,518.64425	−50,366.41237	−769.476579	2011.4196	−0.382553983
	20	−89,946.39638	−38,802.63742	−50,354.16477	−789.594203	1938.111	−0.407404015
	40	−90,253.37684	−39,083.65339	−50,314.9417	−854.78175	1972.1563	−0.433424952
	60	−90,319.32405	−39,168.891	−50,388.66671	−761.766341	1827.078	−0.416931483
	80	−90,833.45732	−39,758.07047	−50,425.3578	−650.029058	1593.2046	−0.408000992
+NPBA RDX	0	−90,046.26317	−38,797.85114	−50,433.27899	−815.133039	1995.4085	−0.408504343
	20	−90,113.00187	−38,932.92598	−50,308.26078	−871.815113	2069.6748	−0.421232898
	40	−90,502.48705	−39,314.25018	−50,324.16339	−864.073482	1977.0356	−0.437055095
	60	−90,772.62685	−39,631.59512	−50,326.1353	−814.896427	1922.7392	−0.423820572
	80	−90,822.96321	−39,709.27646	−50,425.80609	−687.880661	1641.0552	−0.419169728

**Table A2:** The detailed calculated values of interfacial binding energy for varying decomposition degrees of nitrate esters.

Interface Type	Decomposition Degrees of Nitrate Esters/%	*E_total_*/kcal·mol^−1^	*E_matrix_*/kcal·mol^−1^	*E_partical_*/kcal·mol^−1^	*E*_bind_/kcal·mol^−1^	Interface Area/Å^2^	Binding Energy/kcal·mol^−1^·Å^−2^
HMX	0	−90,890.28969	−38,460.5983	−51,495.5587	−934.132689	2894.44	−0.322733478
	20	−88,442.8332	−36,012.92035	−51,445.55238	−984.360465	2851.56	−0.345200685
	40	−85,676.40709	−33,366.17878	−51,325.36956	−984.858756	2848.3569	−0.345763818
	60	−83,114.08306	−30,670.32998	−51,372.57112	−1071.18196	2835.5625	−0.377767007
	80	−79,077.8866	−26,642.32835	−51,318.58435	−1116.973909	2863.3201	−0.390097464
+NPBA HMX	0	−91,156.51333	−38,802.71472	−51,397.30879	−956.489827	2884.7641	−0.331566046
	20	−88,651.03421	−36,296.93009	−51,334.58468	−1019.519441	2869.7449	−0.355264832
	40	−86,993.71519	−34,597.44835	−51,342.2324	−1054.03445	2903.0544	−0.363077747
	60	−83,489.60084	−31,033.66145	−51,347.51526	−1108.424124	2889.0625	−0.383662217
	80	−79,475.03724	−27,255.34556	−51,130.76391	−1088.92778	2661.0122	−0.409215629
RDX	0	−89,946.39638	−38,802.63742	−50,354.16477	−789.594203	1938.111	−0.407404015
	20	−87,633.55242	−36,559.01937	−50,178.94024	−895.592812	2086.5357	−0.429224773
	40	−84,939.81562	−33,738.74097	−50,342.64944	−858.425209	1927.5766	−0.44533909
	60	−82,304.82949	−31,217.79893	−50,236.18095	−850.849603	1878.3009	−0.452988977
	80	−78,458.06411	−27,338.18648	−50,311.15764	−808.71999	1682.6328	−0.480627734
+NPBA RDX	0	−90,113.00187	−38,932.92598	−50,308.26078	−871.815113	2069.6748	−0.421232898
	20	−87,792.49125	−36,584.81986	−50,283.39165	−924.279744	2104.3826	−0.439216587
	40	−86,140.98994	−34,845.85873	−50,373.22266	−921.908551	2011.4196	−0.458337261
	60	−82,643.21283	−31,467.40265	−50,311.74953	−864.060661	1875.258	−0.460768951
	80	−79,133.56185	−27,899.68447	−50,441.20724	−792.670135	1593.2224	−0.497526356
